# A deep learning based NeuroFusionNet approach for automated brain tumor diagnosis from MRI

**DOI:** 10.3389/fninf.2026.1795354

**Published:** 2026-04-16

**Authors:** Omara Mustafa, Salem Alhatamleh, Hamad Yahia Abu Mhanna, Abdallah Almahmoud, Rami Malkawi, Majd Malkawi, Abdel-Baset Bani Yaseen, Hanan Fawaz Akhdar, Hatem Malkawi, Fatimah Maashey, Latifah Alghulayqah, Mohammad Amin

**Affiliations:** 1 Department of Radiology, Korean Medical Center, Lusail, Qatar; 2 Computer Science Department, Faculty of Information Technology and Computer Sciences, Yarmouk University, Irbid, Jordan; 3 Department of Medical Imaging, Faculty of Allied Medical Sciences, Isra University, Amman, Jordan; 4 Department of Allied Medical Sciences-Radiologic Technology, Faculty of Applied Medical Sciences, Jordan University of Science and Technology, Irbid, Jordan; 5 Department of Information Systems, Faculty of Information Technology and Computer Science, Yarmouk University, Irbid, Jordan; 6 Faculty of Medicine, Jordan University of Science and Technology, Irbid, Jordan; 7 Department of Medical Imaging, Faculty of Applied Medical Sciences, The Hashemite University, Zarqa, Jordan; 8 Physics Department, College of Science, Imam Mohammad Ibn Saud Islamic University (IMSIU), Riyadh, Saudi Arabia

**Keywords:** artificial intelligence, brain tumor classification, deep learning, generative adversarial network, MRI image, VGG16

## Abstract

**Background:**

Brain tumor diagnosis from magnetic resonance imaging (MRI) remains a challenging task due to the high variability in tumor appearance and the limitations of manual interpretation.

**Methods:**

To address these challenges, this paper proposes NeuroFusionNet, a deep learning framework for automated brain tumor classification from MRI. The framework integrates GAN-based synthetic image generation with transfer learning using a fine-tuned VGG16 backbone. Real and GAN-generated MRI images are passed through VGG16 to extract discriminative feature representations, which are then used for final classification. To adapt the model to domain-specific MRI characteristics while preserving pretrained knowledge, the last ten layers of VGG16 are fine-tuned and the remaining layers are kept frozen.

**Results:**

The effectiveness of NeuroFusionNet is validated on two publicly available brain MRI datasets. Experimental results demonstrate that the proposed learning framework achieves classification accuracies of 99.05 and 98.75% on the Brain Tumor MRI Dataset and the MRI with Bounding Boxes Dataset, respectively, consistently outperforming several state-of-the-art neural architectures, including VGG16, VGG19, MobileNetV2, DenseNet121, and NASNetLarge.

**Conclusion:**

The results suggest that NeuroFusionNet is effective for the evaluated public MRI datasets; additional external validation is required.

## 1 Introduction

Brain tumors arise from abnormal cell proliferation within the brain or adjacent intracranial structures and may be primary or metastatic. They are classified into two categories: primary, which indicates that they originate in the brain, and secondary, which indicates that they spread from another area of the body. Additionally, types of brain tumors are classified based on their histomorphology, which is determined by their unique molecular characteristics and tissue histology (DeAngelis, 2001). Meningioma is the most common primary CNS tumor overall, whereas glioblastoma is the most common malignant primary brain tumor (Van Meir et al., 2010), they constitute a significant threat to public health. Gliomas can impact any region of the brain and may present as either well-defined or diffusely dispersed tumors.

Gliomas are tumors of the glial cells of the brain, which are used to support the functioning of the nerves inside the brain. These tumors belong to a category called astrocytoma, oligodendrogliomas, brain stem gliomas, and optic gliomas, all of which may be classified as either benign or malignant neoplasms (Louis et al., 2021). The most common type of brain tumor is meningioma, which originates from the meninges, the membrane that encases and protects the brain and spinal cord. Approximately 10–15% of meningiomas are categorized as atypical or malignant, but the rest, constituting 85–90%, are benign. A pituitary adenoma is the commonest tumor of the pituitary gland, the organ controlling the endocrine system. Tumors of the pituitary gland are classified according to their size into pituitary microadenomas (those with a diameter of less than 10 mm), pituitary macroadenomas (greater than 10 mm), and massive tumors (more than 40 mm) (Molitch, 2017).

Research on the classification of brain tumors is complex and continuing. This study aimed to classify several forms of brain tumors using MRI scans. Computed Tomography (CT) and Magnetic Resonance Imaging (MRI) are two prevalent technologies for detecting irregularities in the location, size, or composition of brain tissue (Muhammad et al., 2020). Medical experts favor MRI over other diagnostic methods, and research in this field is increasingly significant. Tissues can be imaged with CT and MRI to determine their dimensions, morphology, and position.

Recent developments in medical AI have increasingly validated machine learning in a spectrum of diseases. For instance, federated learning is the focus of extensive research concerning IoT-based health applications (Campos et al., 2022), while ensemble approaches have generated great results in coronary artery disease detection (Sapra et al., 2021). Supervised learning methods, too, have been efficiently used in the classification of neurological conditions, such as migraine (Gulati et al., 2022). The above studies illustrate that AI is expected to play an increasingly important role in the health domain, thus spurring more investigations into deep-learning architectures, mainly NeuroFusionNet, for complex diagnostic problems. In the present study, despite the availability of recent architectures such as ResNet50 and Vision Transformers, VGG16 was selected as a backbone due to its stability, simplicity in transfer learning, and demonstrated strong performance in multiple medical imaging tasks. To counter class imbalance, the minority tumor classes were oversampled, and class weighting in training was implemented. A mean squared error (MSE) loss function was used to train the GAN component to enhance the critical features corresponding to MRI images for classification. As for transfer learning, this was achieved by unfreezing the last ten layers of VGG16, with loss convergence monitored through validation loss tracking and early stopping. For completeness and reproducibility, a full architectural block diagram has been inserted to illustrate GAN-based feature enhancement combined with transfer learning and classification layers.

This study aims to improve the care of patients with brain tumors and increase the accuracy of diagnosis by leveraging deep learning models. The main contributions of this study are as follows:

- A new hybrid method, VGG16 and generative adversarial networks (GANs) (NeuroFusionNet), is presented to identify different types of brain tumors and distinguish them from healthy tissue.
- To improve brain tumor classification performance, the suggested model incorporates deep transfer learning.
- The NeuroFusionNet was compared with VGG16, VGG19, MobileNetV2, DenseNet121, and NASNetLarge under a consistent experimental setting.
- To improve generality and resilience, the model uses data augmentation techniques, fine-tuning strategies, and an ideal learning rate schedule.

## 2 Methods

**Figure 1:**
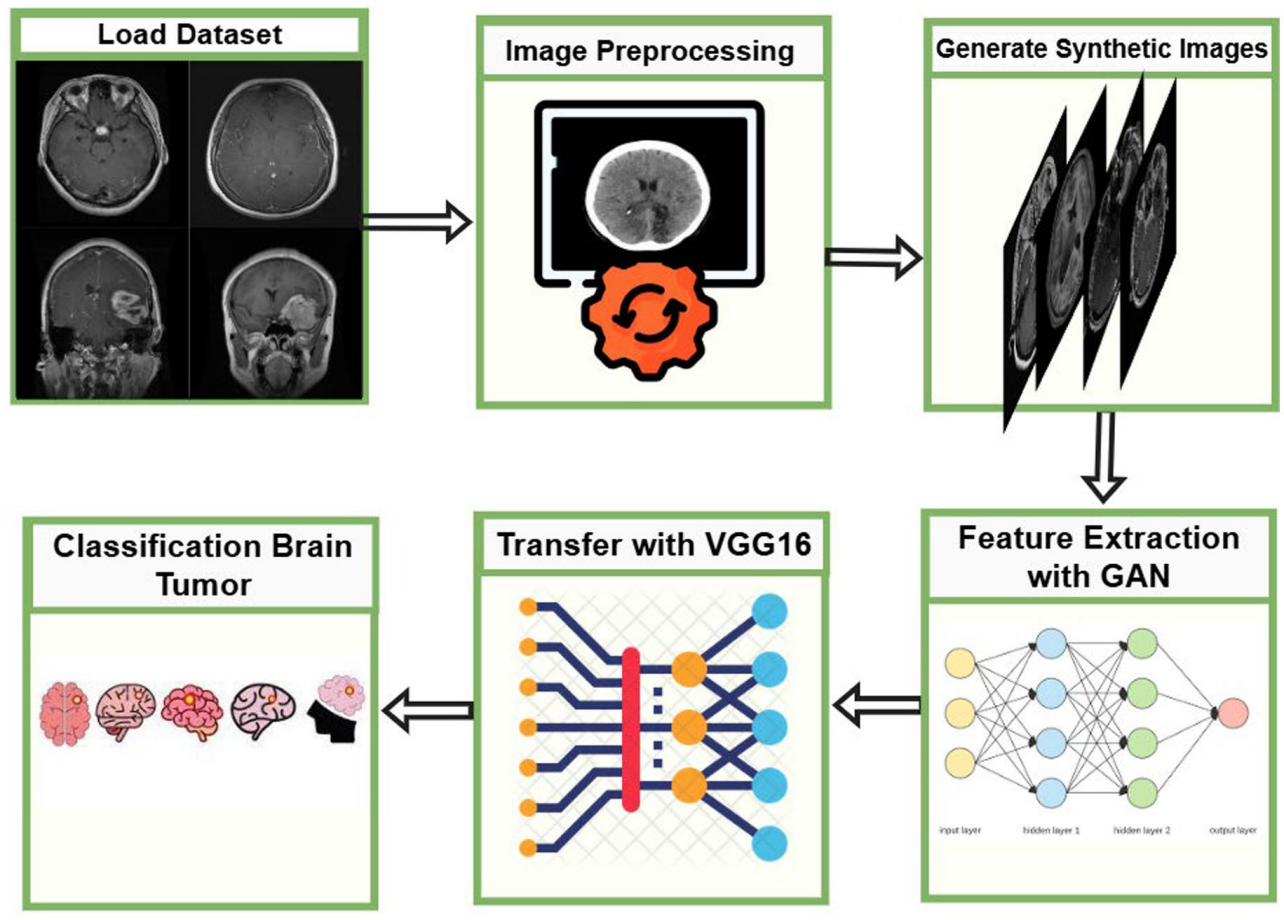
High-level overview of the proposed NeuroFusionNet workflow for brain tumor classification.

The proposed NeuroFusionNet framework combines GAN-based synthetic image generation with transfer learning using a VGG16 backbone to classify brain MRI images into four classes: glioma, meningioma, no tumor, and pituitary tumor, as shown in Figure 1. Real and GAN-generated MRI images are passed through the VGG16 backbone to extract feature representations, which are then combined before the final classification layer. The last ten layers of VGG16 were unfrozen for fine-tuning, while the remaining layers were kept frozen to preserve pretrained ImageNet representations. Classification was performed using a custom classifier head composed of Global Average Pooling, dense layers, batch normalization, and dropout, followed by a softmax output layer. Adam optimization with cosine decay learning-rate scheduling, early stopping, and ReduceLROnPlateau was used during training.

### 2.1 Dataset

**Figure 2:**
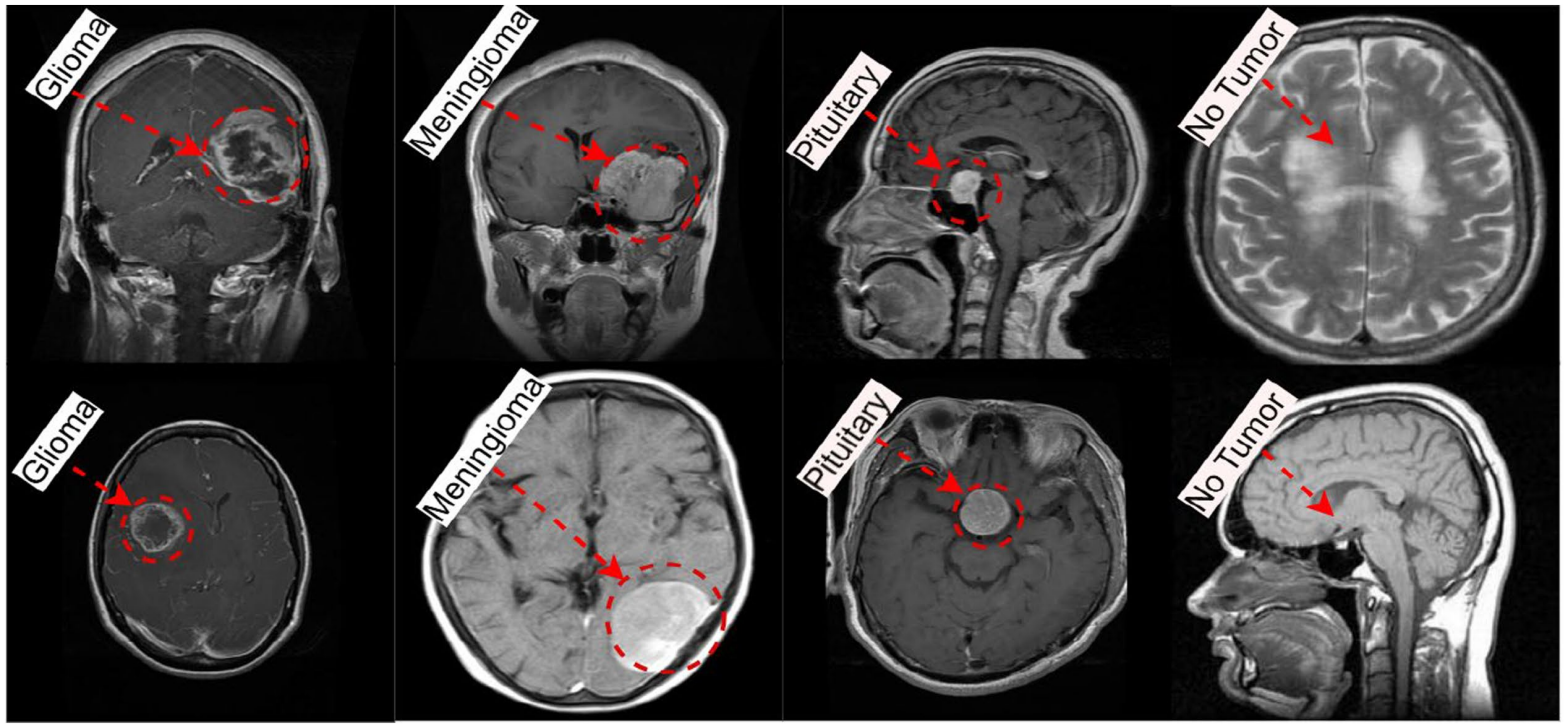
Sample in the data brain tumor MRI dataset and MRI for brain tumor with bounding boxes.

The methodology was evaluated using two datasets: the Brain Tumor MRI dataset (Esfandiari, 2025), with selected squares, including a 7,153 MRI images, and another brain tumor MRI with boundary boxes (Sorour, 2025), consisting of 5,249 MRI images. Both datasets comprise four categories: glioma, pituitary, meningioma, and No tumor, as illustrated in Figure 2. Table 1 illustrates the allocation of classes for both datasets.

**Table 1:** Number of images and category: brain tumor.

Dataset	Case	Number of images
Brain tumor MRI dataset	Glioma	1,621
	Meningioma	1775
	No tumor	2000
	Pituitary	1757
	Total	7,153
Brain tumor MRI with boundary boxes	Glioma	1,289
	Meningioma	1,589
	No tumor	811
	Pituitary	1,560
	Total	5,249

All data used in this study were obtained from publicly available databases, and their usage did not require patient consent or institutional authorization, as they are publicly available and de-identified datasets, in accordance with applicable ethical guidelines

### 2.2 Preprocessing

**Figure 3:**
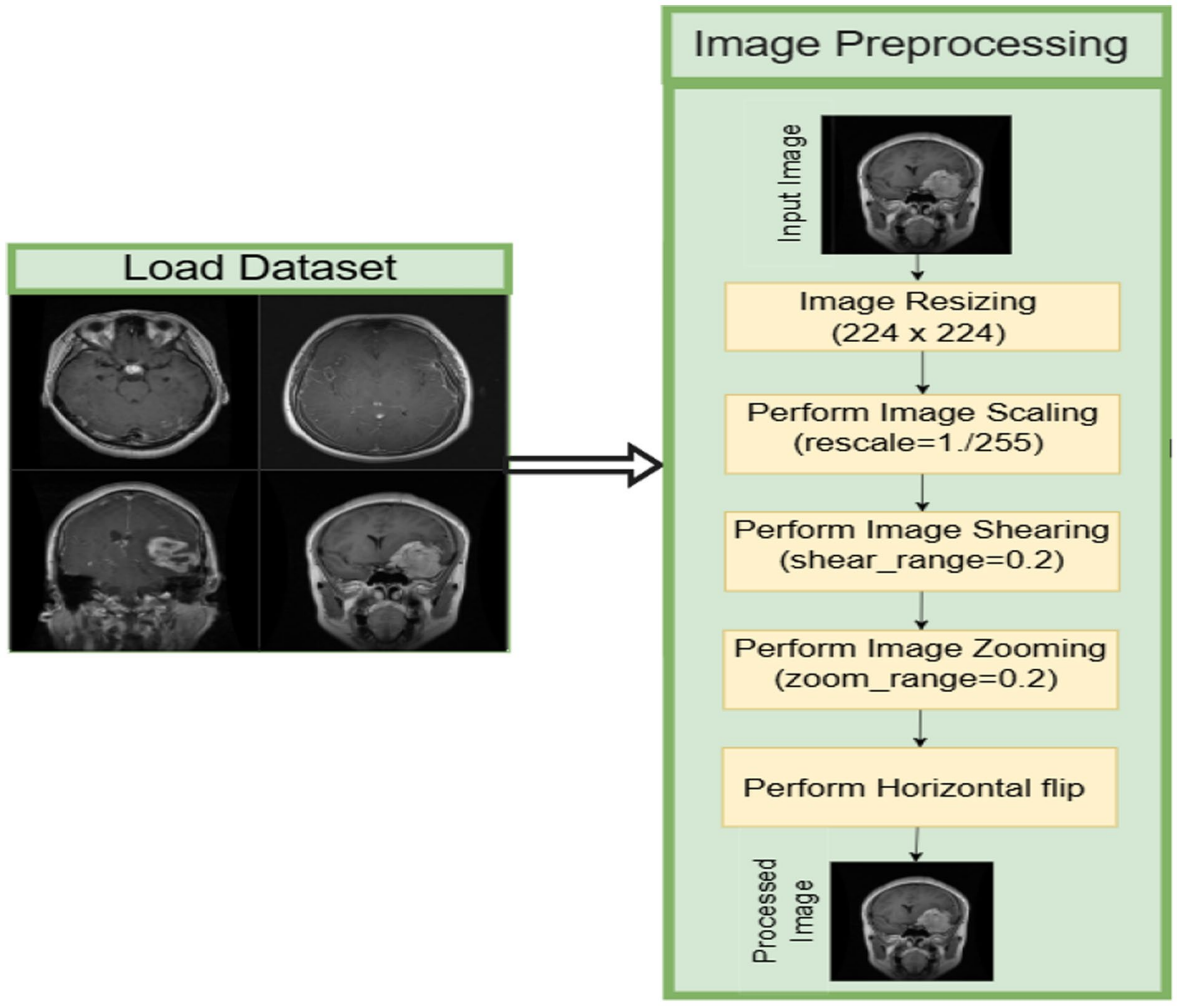
Dataset preprocessing steps.

In this part of the methodology, the preprocessing pipeline has been discussed in detail to address class imbalance and augment MRI images from two Kaggle datasets: the Brain Tumor MRI Dataset and the MRI for Brain Tumor with Bounding Boxes. Class distributions in the original datasets were first analyzed to determine which of the four tumor types was imbalanced (Brummer et al., 1993). For instance, the classes were resampled using the random oversampling of minority classes via duplication until they had equal representation across all classes. The newly created dataset was saved to a new directory, including the original images and replicated images (Huang et al., 2023). All images were resized to the same dimensions of 224 × 224 pixels, meeting requirements for input to deep learning models like VGG16 for computational efficiency and uniformity during training. To ensure proper evaluation, the dataset was first divided into training (80%), validation (10%), and testing (10%) subsets. Data augmentation and balancing techniques were subsequently applied to improve class representation and enhance model generalization. Care was taken to maintain separation between training and evaluation processes, ensuring that model performance reflects its ability to generalize to unseen data, as illustrated in Figure 3. More importantly, to improve the performance of the model, data augmentation was performed with the Image Data Generator in TensorFlow, in which random transformations were applied, including rotations, zooms, shearing, shifting, and flips across a horizontal axis that represented variability in the real world and would create different representations of MRI images (Atienza, 2020).

### 2.3 Methods of transfer learning

**Figure 4:**
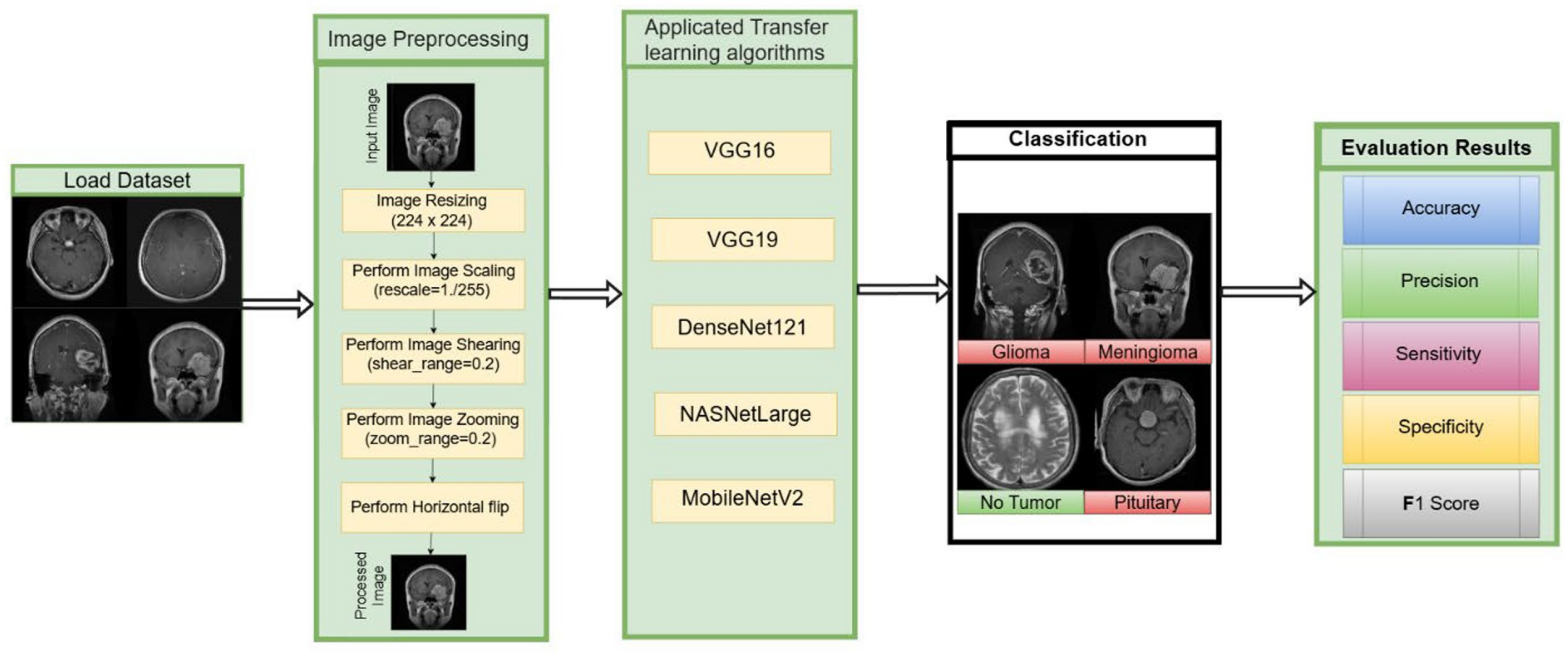
The overall workflow methods of transfer learning in this study.

Pre-trained models are deep learning models that have been created and trained on extensive datasets (Zhuang et al., 2020). As a result, they can extract vast amounts of pertinent data from the input. These models provide a strong foundation for applications such as image classification, language translation, and object detection. Figure 4 illustrates the workflow of transfer learning adopted in this study. Furthermore, fine-tuning pre-trained models can significantly enhance performance, especially when working with limited or domain-specific datasets, while also reducing training time and computational cost. In this study, the proposed model is compared with five pre-trained architectures: VGG16, MobileNetV2, VGG19, DenseNet121, and NASNetLarge.

### 2.4 Data augmentation generators with GAN

In this manner, both data augmentation methods and the so-called Generative Adversarial Network (GAN) are implemented separately, which enhances the training process and performance of the deep learning model. Here is how these two methods complement each other: they can obtain a more diverse and representative dataset, which enables the model to perform even better on data it had not encountered earlier.

**Figure 5:**
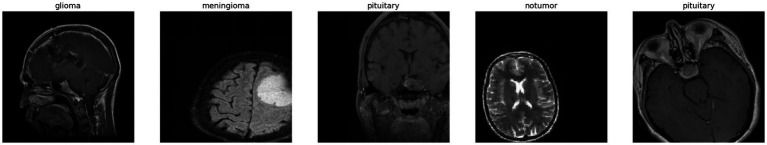
Sample from data generator.

As per the explanation, augmentation is done on training images, capturing crops of an image through the Image Data Generator class (Godishala et al., 2022). The generator utilizes transformation techniques including rotation, zoom, width/height shift, and even horizontal flipping of the target images. These modifications help the model during the training phase by trying to wipe out memorization of the distinct patterns within the images and instead instill generalized features across the distinct versions. By dynamically generating these augmented images during training, the model effectively gets a different version of the data in every epoch cycle. Such might help to counter overfitting by encompassing a greater variety of data. As shown in the sample from the Data Generator Figure 5.

**Figure 6:**
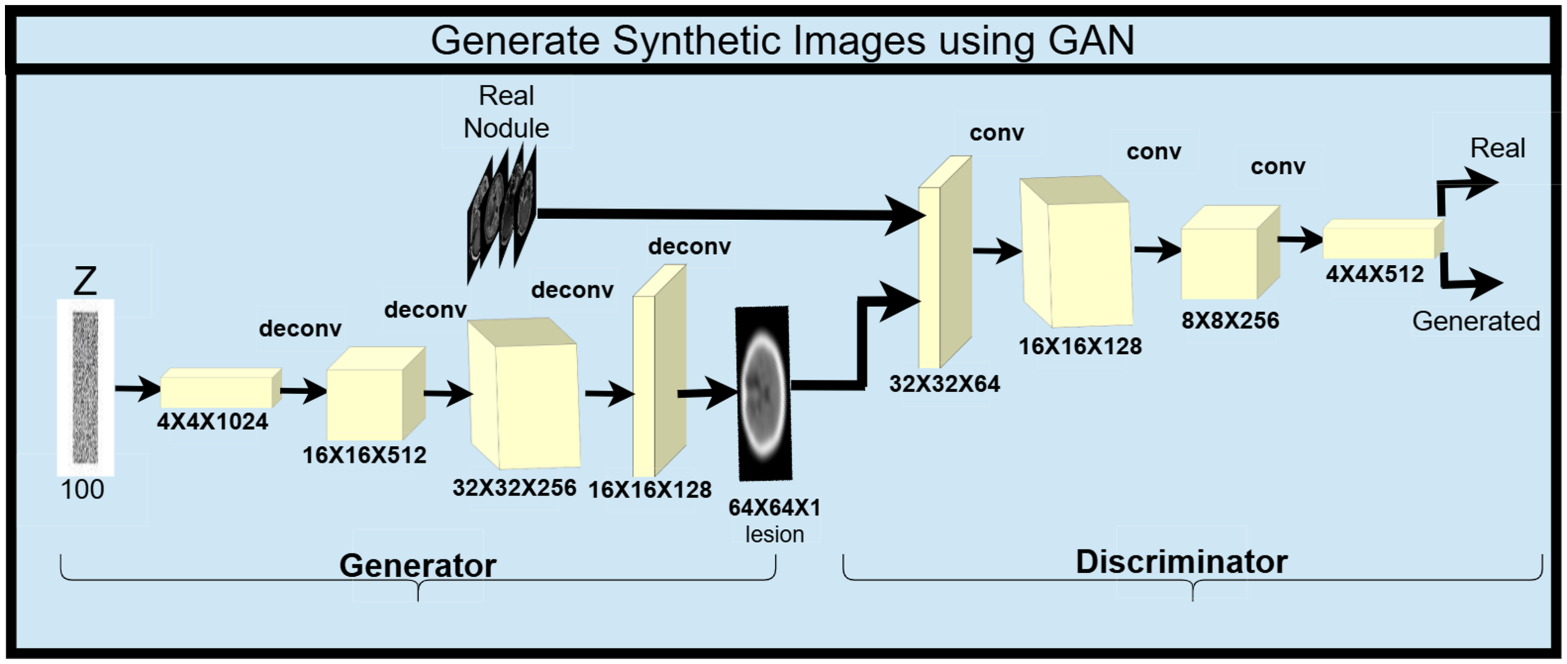
The overall workflow of synthetic images using GAN.

Besides, a Generative Adversarial Network (GAN) is also used to perform this task and to expand the dataset even more. The GAN comprises two important models – a Generator and a Discriminator (Mardani et al., 2018). The generator generates images from random latent vectors (random noise) by feeding them to transposed convolution layers for creating realistic-looking images. These images are called synthetic images and are aimed at increasing the variety of original training data. On the other hand, the discriminator is made to tell apart real images of the dataset from fake images produced by the generator. With the fake ones, convolutional layers and Leaky ReLU activations analyze the images. The discriminator evaluates the quality of the generated images to enhance the generation of novel images by the generator. As shown in the Overall Workflow GAN Figure 6.

To produce a dataset that is both larger and more diverse, the combination of methods of data augmentation and GAN-generated images is employed in this approach (Han et al., 2019). Additional variants of original data are contributed with augmented images, whilst GAN images add completely novel data points. All these techniques together improve the training process by making sure that the model is trained with a rich and varied set of images. Furthermore, the data generators used for both augmentation and synthetic image creation ensure that images are adequately preprocessed, scaled, and shuffled before being input into the model for a seamless and efficient training process. In GANs, a least-squares adversarial loss was used for the discriminator to distinguish between real and synthetic images as shown in (Equation 1):


$$L_{D}=\frac{1}{N}\sum_{i=1}^{N}[D{\left(x_{i}-1\right)}^{2}+D{\left(G(Z_{i})\right)}^{2}]$$ (1)

As an ablation study for the evaluation of the influence of GAN-generated data on classification performance, two distinct versions of the model NeuroFusionNet were trained: One with traditional augmentation only and the other with both traditional augmentation and GAN-generated samples. The results showed a classification accuracy gain of 1.72% on the Brain Tumor MRI Dataset and 1.41% on the MRI with Bounding Boxes Dataset when GAN-generated images were utilized. This gain is credited to the enrichment of minority classes with the synthetic data and variability that allows better feature learning by the model. This work improved the generator by increasing epochs, fine-tuning the discriminator feedback using label smoothing, and manipulating noise vector sampling to yield or induce more realistic image structure. These improvements improved classification accuracy of synthetic data diversity and realism, leading to increased robustness and generalization of the classification model.

To ensure class balance, synthetic images were generated for minority classes until each class reached the same number of samples as the majority class within the training set. The generated images were combined with real images to form a balanced dataset, with an approximate real-to-synthetic ratio determined dynamically based on class distribution.

The GAN was trained using a least-squares adversarial loss, and stability was improved through techniques such as label smoothing and controlled noise vector sampling. Although no explicit quantitative metrics such as Fréchet Inception Distance (FID) were computed, visual inspection and training stability were used as practical indicators of image quality.

### 2.5 Feature extraction with GAN

The proposed technique of feature extraction using a Generative Adversarial Network (GAN) works in an advanced way: the generator model of the GAN is used to generate synthetic images, from which discriminative features are extracted that can be utilized in the training of a classifier, or for the enhancement of an existing model (Abdollahi et al., 2020). It is a two-pronged approach-increasing the dataset size and permitting the model to learn useful and diverse features from both synthetic and real data.

A GAN model is trained using the min-max game between a discriminator D and a generator network G in the original GAN formulation (Goodfellow et al., 2014). The GAN aims to approximate a probability distribution function, which is supposed to be the source of some data. The generator-discriminator min-max game’s goal function can be expressed as follows as shown in (Equation 2):


$$\min_{G}\max_{D}E_{x\sim P_{r}(x)}[\log D(x)]+E_{Z\sim P_{Z}(Z)}[\log(1-D(z))]$$ (2)

When given a certain random latent vector input, the GAN’s generator creates synthetic images (You et al., 2022). These random latent vectors act as noise inputs, inputs that the generator converts into images at a resolution of 224 × 224 pixels, like the samples it was trained on. The artificial images generated by such research can then be supplemented into samples for training purposes to get a better model. In cases where the original dataset is small, these provide a way to improve model learning with a more diverse range of examples.

Construction for this custom generator architecture is in principle deep convolutional, in keeping with the spirit of DCGAN. The initial ingredient is a fully connected input layer that reshapes the noise vector into a 7 × 7 × 256 tensor and follows the series of transposed convolutional layers to upsample the resulting tensor into high resolution images. More details of the structure are as follows. Input layer a 100D latent vector is projected and reshaped as shown in (Equation 3):


$$h_{0}=\text{ReLU}(\text{BathNorm}(W_{0}+b_{0}))\;\;\;\;h_{0}\in {\mathbb{R}}^{7\times 7\times 256}\;$$ (3)

Upsampling layers three Conv2DTranspose layers upsample the tensor to 14 × 14, 28 × 28, and 56 × 56 resolutions with decreasing filter sizes (128, 64, 32), each followed by batch normalization and ReLU activation as shown in (Equation 4):


$$h_{i}=\text{ReLU}(\text{BathNorm}(\text{Conv}2\text{DTranspose}(h_{i}-1)))\;$$ (4)

Output layer a final Conv2DTranspose layer generates a 224 × 224 × 3 RGB image as shown in (Equation 5):


$$\hat{x}=\tanh(\text{Conv}2\text{DTranspos}e_{\text{output}}(h_{3}))\;$$ (5)

Then the process will continue by generation of synthetic images, where the first step will involve introducing the synthetic images into a pretrained feature extraction backbone to extract relevant features from each generated image. The synthesized features would then allow the model to capture common modes in the data that may not be present in the actual training set. Once trained, the generator generates a variety of synthetic images, which, in union with real data, enable the model to balance some of the broader patterns and improve generalization. In a post-processing stage, features from these synthetic images are extracted using a pretrained backbone (VGG16) and fused with features from real images before passing into the final classification layer of NeuroFusionNet.

**Figure 7:**
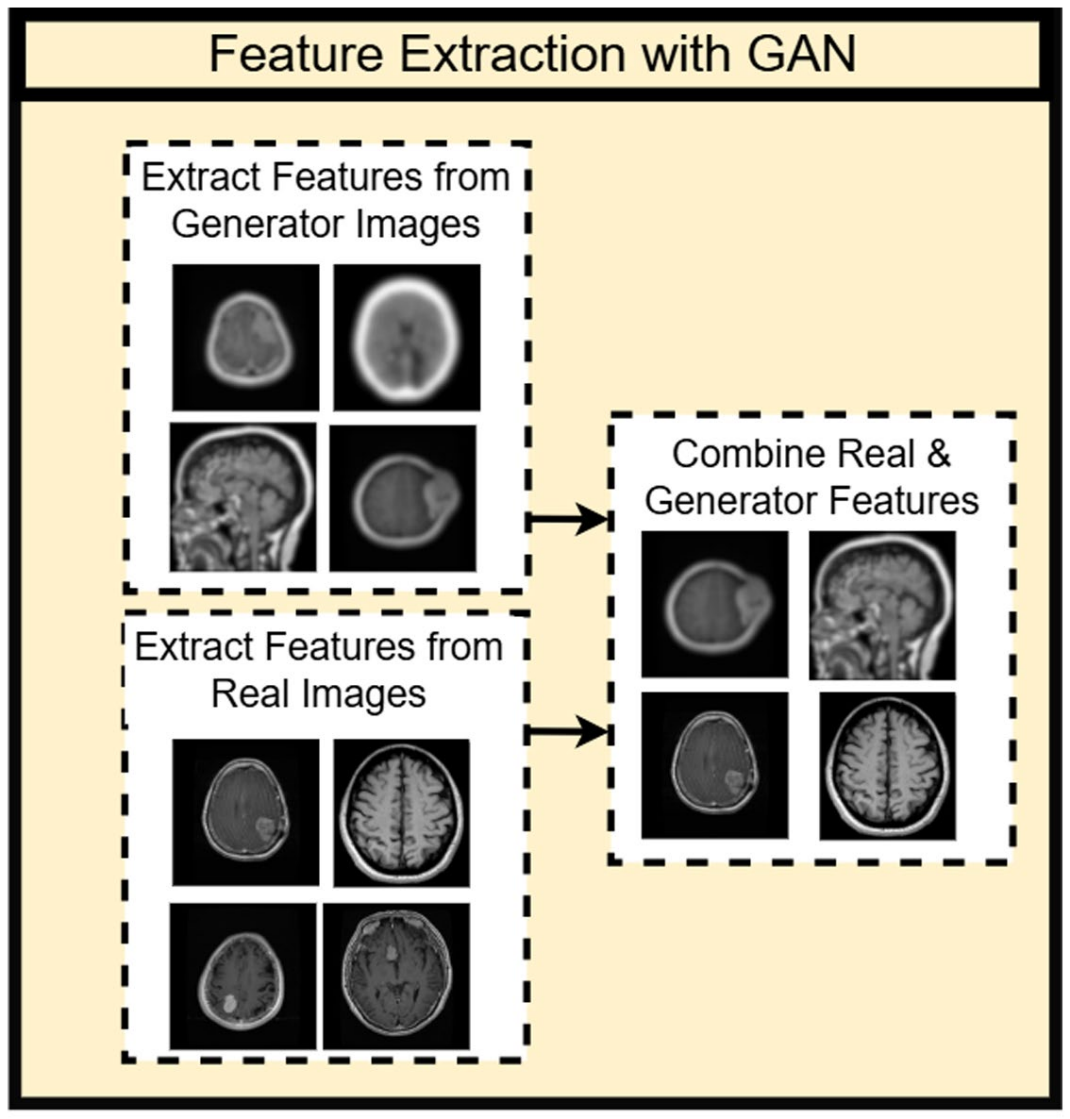
Feature extraction with GAN.

This feature extraction framework, assisted by GAN-generated data, provides more diversity to the dataset and teaches the model from both real-world and synthetic examples, which makes improvements in generalization. Generation of significant features leads the model to learn a comprehensive representation of wider data diversity, which eventually leads to better performance in various tasks like classification and segmentation. To put it briefly, feature extraction with GAN can help augment the dataset by creating synthetic images, contributing to the higher variability of the data, which in turn produces a better model performance. This is how the mixture of real and synthetic data helps create more generalized decisions that can recognize more complex patterns and improve the accuracy of predictions. As shown in Figure 7.

### 2.6 Transfer learning with VGG16

Transfer learning utilizes the already trained VGG16 model (Barman et al., 2024) to enable the re-utilization of features learned from (GAN) due to its prowess in capturing very complex and hierarchical features of spatial properties. The classification process deals with the identification of four tumor types: glioma, meningioma, no tumor, and pituitary. Here, the VGG16 model is used along with features extracted from GANs. The generator model of the GAN generates synthetic images from random latent vectors, which are then fed into the VGG16 model. These synthetic images offer a rich set of features that complement the original dataset. So, after proving the extracted features that represent the characteristics of both the original and synthetic images for the subsequent layers of the transfer learning pipeline.

**Figure 8:**
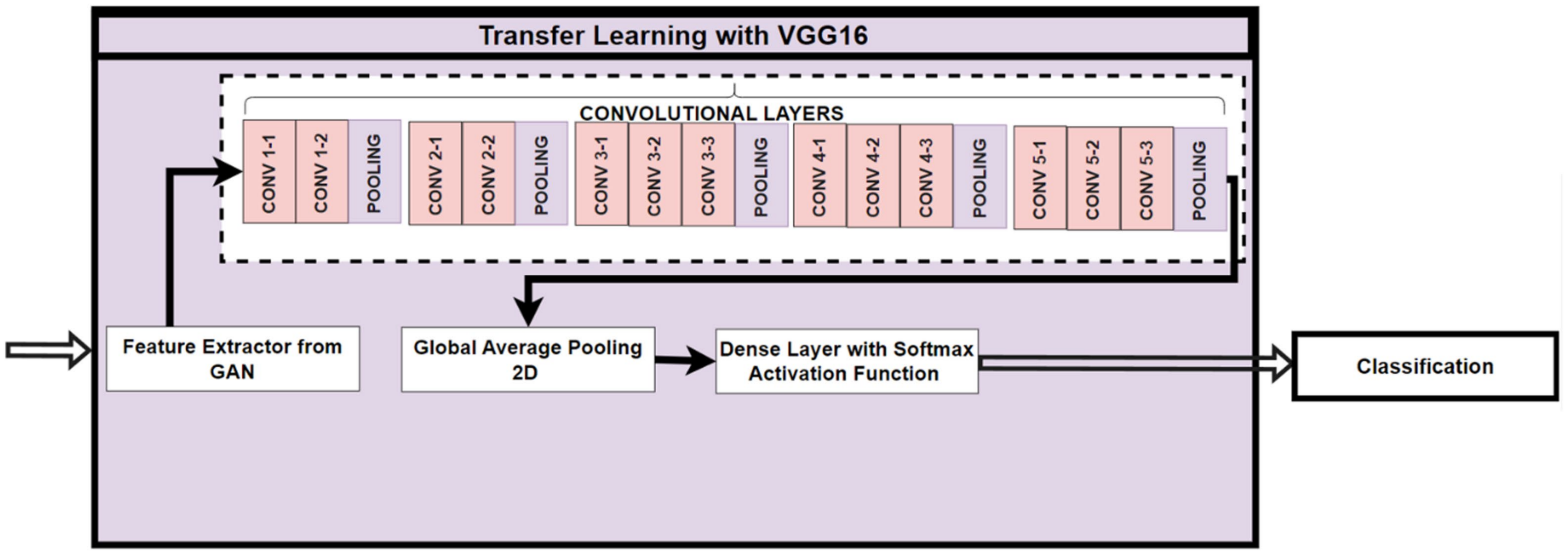
The overall workflow: transfer learning with VGG16.

The customized classifier is employed on the uppermost layers of the VGG16 model and transformed into a classifier specific to the task; it also adds Dense layers, batch normalization, and dropout. As a result, these images can be classified into four categories of tumors. While the lower layers of the VGG16 model remain frozen, retaining the advantages of pre-trained features, the upper layers are fine-tuned to adjust the model for the new task. This fine-tuning of the model allows it to reproduce better the high-level feature representation from the combined dataset of real and synthetic images. In this paradigm, GAN-based feature extraction competes with transfer learning to fortify the generalization ability of the model to the dataset. The rich features achieved through VGG16 are made even richer when they are provided with different angles and improvements of synthetic variations from the GAN approach. This creates synergy much grander with GAN and VGG16 in their robustness for classifying the minor patterns, getting a level of accuracy in classification on the brain tumor dataset. As shown in the overall workflow Figure 8.

### 2.7 Proposed model: NeuroFusionNet

NeuroFusionNet has been named for the model’s core purpose and its design. The prefix “Neuro” represents its focus on neuroscience and neuroimaging, as it is specifically fashioned to process and segment those kinds of MRI images to detect and classify brain tumors. The word “Fusion” refers to the extraordinary combination of the technologies embedded in the model. This approach combines the high-dimensional feature extraction from a GAN-based generator and the domain-specific knowledge from a pre-trained VGG16 model in which the generator and the model are synthesized in such a way that the results appear to be both robust and accurate. Besides the technological characteristics, the synergistic combination of generative modeling and transfer learning aspects is also described by this fusion, and this mechanistic arrangement leads to a holistic and more informative depiction of tumor traits. At the very end of the term, the term “Net” is the one that describes that it is a neural network that constitutes the main part of this solution. The name of NeuroFusionNet encompasses the model’s cutting-edge, interdisciplinary approach to diagnosing and classifying brain tumors, as the model is of crucial importance for brain tumor detection.

The model uses a GAN to extract features and integrates them with a transfer learning framework to leverage pre-trained knowledge. The process begins with a GAN generator, which processes brain MRI images to create high-quality, multidimensional features. The generator network is shaped from layers of convolution, batch normalization, and up-sampling, and it is designed to adjust image features, which are the most important for the differentiation of tumor types. These extracted features, which are more informative and understandable, are used as input data for the process of transfer learning, where we use the VGG16 model. VGG16 provides a robust foundation by using learned filters that capture general image patterns including edges and textures, which are later adjusted to the tumor classification task. To conduct fine-tuning on VGG16, we limit the last ten layers to be the only ones trainable so that the network retains the learned features but evolves as required. After the primary architecture, the VGG16 outcome is reshaped by Global Average Pooling (GAP), dense layers, batch normalization, and dropout regularization (Hsiao et al., 2019), thus, the model will be enabled to accumulate shared knowledge while at the same time fighting overfitting. The final layer is a softmax layer, which generates probabilities for the four tumor classes. The categorical cross-entropy loss function guides the training process (Ho and Wookey, 2019), and it is given by as shown in (Equation 6):


$$L=-\frac{1}{N}\sum_{i=1}^{N}\sum_{c=1}^{C}y_{i,c}\log({\hat{y}}_{i,c})$$ (6)

Where $y_{i,c}$ is the true label (one-hot encoded) for the class c of sample ${\hat{y}}_{i,c}$ is the predicted probability for the class c , N is the number of samples and C is the number of classes. The purpose of this loss is to make sure that the predicted probabilities of the classes are in close agreement with the true labels. The optimization process in NeuroFusionNet uses plans that are even more fine-tuned by adding a Cosine Decay Learning Rate Scheduler (Konar et al., 2020), which automatically scales the learning rate $n_{t}$ of every epoch *t*, following a formula as shown in (Equation 7:


$$n_{t}=n_{0}*\frac{1+\cos(\frac{\mathrm{\pi t}}{T})}{2}$$ (7)

**Figure 9:**
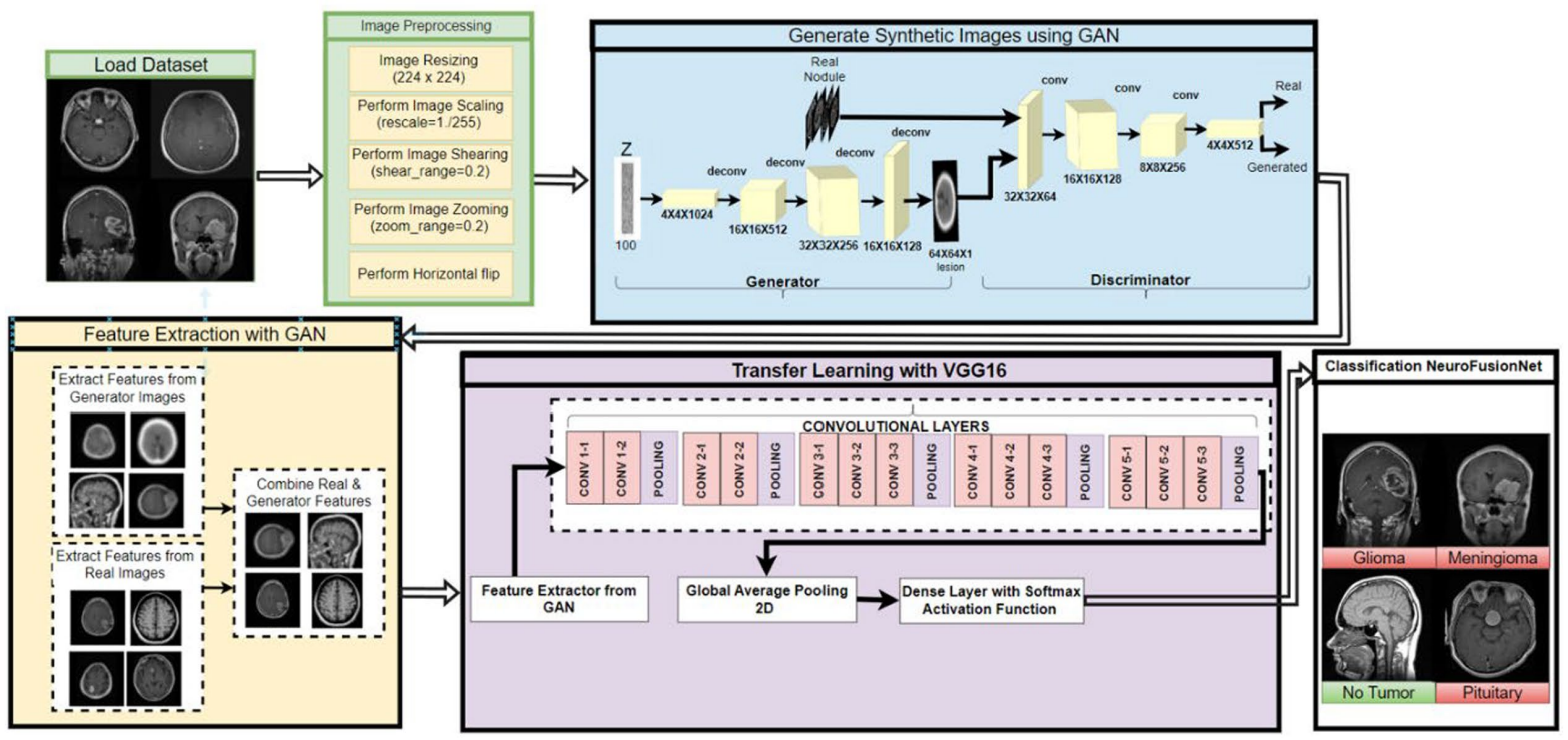
Detailed architecture of the proposed NeuroFusionNet framework, including GAN-based feature extraction and VGG16-based classification.

Where $n_{0}$ signifies the initial learning value and T is the total number of decay steps epochs. This is the method that vouches for the gradual drop in the learning rate, facilitating more significant adjustments at the beginning of the training and smaller but more accurate corrections as the model converges. As an added measure, callbacks such as Early Stopping done with the help of Reduce LROn Plateau can stop training when the validation loss no longer decreases, prevent overfitting, and decrease the learning rate even more when the model’s performance is stagnant (Thakur et al., 2024). One of the methods to monitor the model’s performance during training is to use accuracy scores and a confusion matrix to see class-wise predictions. NeuroFusionNet’s balance of robust feature extraction and proper classification is achieved by combining the generative capabilities of the GAN, the representational power of VGG16, and the optimization techniques, making it a perfect tool for brain tumor diagnosis. As shown in the Overview Figure 9.

### 2.8 Evaluation proposed method

NeuroFusionNet, the methodology for the detection of brain tumors, looks at various indicators that measure the model’s performance, including but not limited to accuracy, precision, sensitivity, specificity, and F1 score. Every single parameter is valuable to determine the model’s capability of providing accurate diagnosis and clinical applicability. The next paragraphs give detailed descriptions and definitions, as well as equations, a discussion of context, and the relationship of all these modules to the classification of Glioma, Meningioma, No Tumor, and Pituitary cases.

*TP (True Positives)*: Cases that the model has diagnosed properly, Glioma, Meningioma, or Pituitary tumors.

*TN (True Negatives)*: Situations in which the model is correct and classifies no tumor images.

*FP (False Positives)*: Instances of a healthy brain image (No Tumor) are misclassified as one of the tumors.

*FN (False Negatives)*: Cases a “brain tumor” that is misclassified as “No Tumor.”

Accuracy is defined as the proportion of correctly classified samples that takes the total occurrence of the correct predictions (both positive and negative) and compares it to the whole number of predictions. It unfolds the full information about the model’s overall effectiveness, particularly in differentiating accurately between Glioma, Meningioma, Pituitary tumors, and no tumor cases as shown in (Equation 8).


$$\text{Accuracy}=\frac{\mathrm{TP}+\mathrm{TN}}{\mathrm{TP}+\mathrm{TN}+\mathrm{FP}+\mathrm{FN}}$$ (8)

The provision of proportion of correctly identified positive cases to the total number of positive results determines Precision. High precision leads to a reduction in false positives, which is vital in disease categorization and reduction of the number of incorrect cases diagnosis in healthy patients. as shown in (Equation 9).


$$\text{Precision}=\frac{\mathrm{TP}}{\mathrm{TP}+\mathrm{FP}}$$ (9)

Sensitivity (Recall) is a way to assess the model’s ability to accurately identify the true positive cases, which, in turn, tells us how well it detects the Glioma, Meningioma, and Pituitary tumor as shown in (Equation 10).


$$\text{Sensitivity}=\frac{\mathrm{TP}}{\mathrm{TP}+\mathrm{FN}}$$ (10)

Specificity is testing the model’s capacity to correctly tell No Tumor cases; it lowers the chance of false positives. High specificity is extremely important since wrong classification is very common; for example, healthy images can be classified as images of tumors, which can lead to a lot of unnecessary treatments and interventions as shown in (Equation 11).


$$\text{Specificity}=\frac{\mathrm{TN}}{\mathrm{TN}+\mathrm{FP}}$$ (11)

The F1 Score is the harmonic mean of Precision and Sensitivity. Using the F1 Score, the classifications of different tumor types and No Tumor cases can be balanced, and especially when the data distribution is imbalanced this metric is even more helpful as shown in (Equation 12).


$$F1\;\text{Score}=2*\frac{\text{Precision}*\text{Sensitivity}\;}{\text{Precision}+\text{Sensitivity}}$$ (12)

With the incorporation of such evaluations, NeuroFusionNet provides a reliable classification for images about Brain Tumor categories, i.e., Glioma, Meningioma, Pituitary, and No Tumor cases, contributing to a crucial step in medical diagnostics.

### 2.9 Computing environment

The average training time per epoch was approximately 42 s. A PC with Windows 11 Pro, 16 GB of RAM, a 12th-generation i7-12700k 3.50 GHz processor, and an NVIDIA GPU RTX 4060 Ti was used to complete this task.

## 3 Result

A brain tumor MRI (Esfandiari, 2025) and a brain tumor MRI with border boxes (Sorour, 2025) were used in the study to assess the NeuroFusionNet model. Both datasets include image classes for normal tissues and pituitary tumors, gliomas, and meningiomas. Split is used in this work to separate the dataset an 80% for training, 10% for validation, and 10% for testing. To ensure a fair and consistent comparison, identical hyperparameter settings were applied across all models, including the proposed NeuroFusionNet and the baseline architectures. These parameters were selected based on commonly recommended configurations from official implementations and relevant literature. In addition, all models were trained using the same data splits, preprocessing pipeline, and evaluation protocol to eliminate any bias introduced by unequal experimental conditions. Table 2 presents the hyperparameter configuration used for training all models. A learning rate of 0.0001, batch size of 8, and 50 training epochs were selected to achieve stable convergence while maintaining computational efficiency, particularly for high-resolution MRI images. The Rectified Linear Unit (ReLU) activation function was used for intermediate layers, while the Softmax function was applied in the output layer for multi-class classification. The Adam optimizer and a dropout rate of 0.5 were employed to improve generalization and reduce overfitting.

**Table 2:** Unified hyperparameter configuration used for training all models.

Hyperparameter	Value
Image size	(224,224)
Batch size	8
Learning rate	0.0001
Epochs	50
Activation function	Rectified linear unit (ReLU)
Dropout	0.5
Optimizer	Adam
Output activation function	Softmax

### 3.1 Comparing different models

#### 3.1.1 Comparison of models and results on the brain tumor MRI dataset

**Figure 10:**
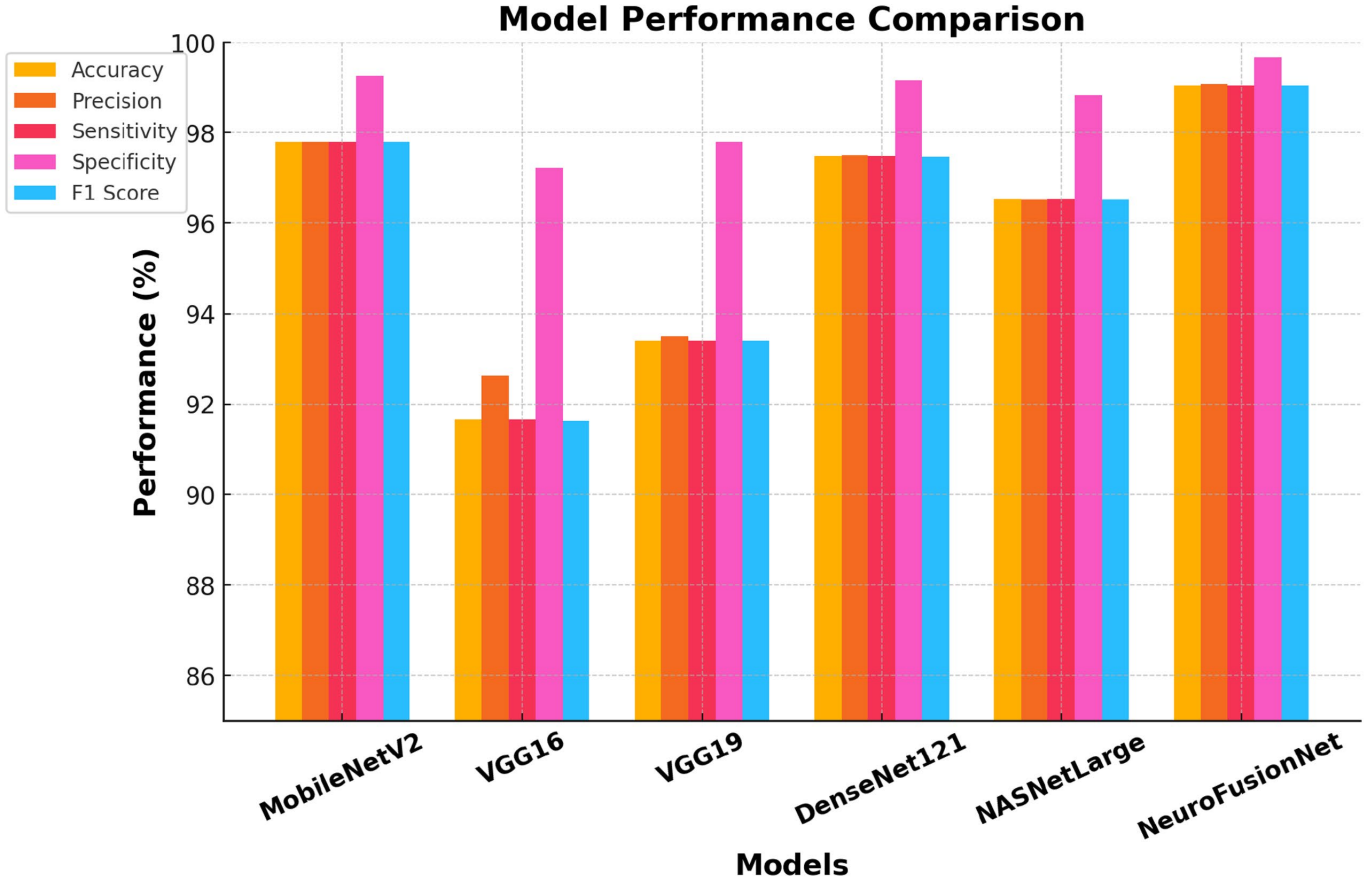
Comparison of performance metrics of different models using the brain tumor MRI dataset.

Initially, five pre-trained models were utilized on two datasets. The identical hyperparameter settings were employed to evaluate each of these models. The efficacy of the trained models was compared to that of the proposed hybrid model, NeuroFusionNet, which employs the pre-trained VGG16 model for transfer learning and GANs for feature extraction. This study effectively utilizes both advanced representations of medical imaging data and the enhancement of synthetic features. On the Brain Tumor MRI Dataset, NeuroFusionNet achieved 99.05% accuracy, 99.07% precision, 99.05% sensitivity, 99.68% specificity, and 99.05% F1 score, as presented in Table 3 and Figure 10. The VGG16 model ranked last, exhibiting the lowest accuracy (91.66%), sensitivity (91.66%), specificity (97.22%), and F1-score (91.63%). Furthermore, a comparison was conducted between two models, MobileNetV2 and DenseNet121, revealing comparable accuracy levels of 97.79% for MobileNetV2 and 97.48% for DenseNet121. The proposed NeuroFusionNet model outperformed the evaluated baseline architectures.

**Table 3:** Performance evaluation on the brain tumor MRI dataset.

Model	Accuracy	Precision	Sensitivity	Specificity	F1 score
MobileNetV2	97.79%	97.79%	97.79%	99.26%	97.79%
VGG16	91.66%	92.63%	91.66%	97.22%	91.63%
VGG19	93.39%	93.51%	93.39%	97.79%	93.39%
DenseNet121	97.48%	97.50%	97.48%	99.16%	97.47%
NASNetLarge	96.54%	96.52%	96.54%	98.84%	96.52%
NeuroFusionNet	99.05%	99.07%	99.05%	99.68%	99.05%

**Figure 11:**
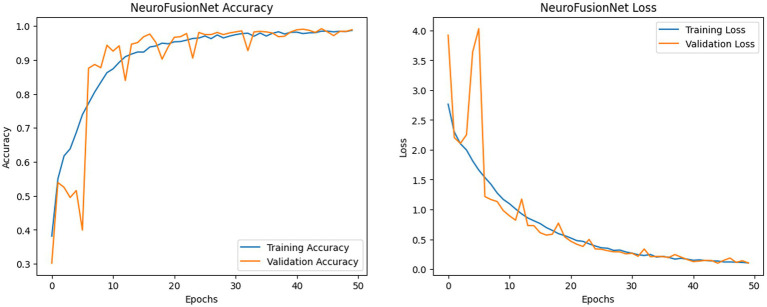
NeuroFusionNet’s training and validation metrics (accuracy and loss) on the brain tumor MRI dataset.

**Figure 12:**
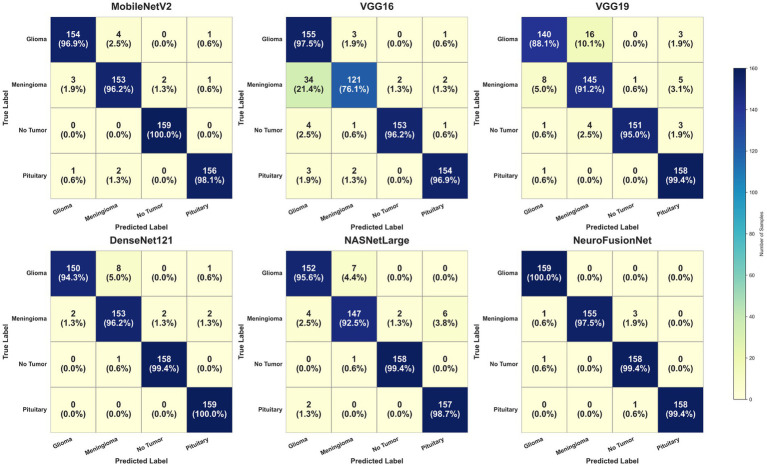
Using the brain tumor MRI Dataset, confusion matrices for each model are compared.

Figure 11 illustrates the enhanced accuracy and diminished error of the proposed model, NeuroFusionNet, during the training and validation phases. The model achieved 99.05%. An enhanced approach to model comparison involves the analysis of confusion matrices. A compilation of both precise and imprecise estimations is referred to as a confusion matrix. Figure 12 presents the outcomes of the confusion matrices.

#### 3.1.2 Comparison of models and results on MRI for brain tumor with bounding boxes dataset

**Figure 13:**
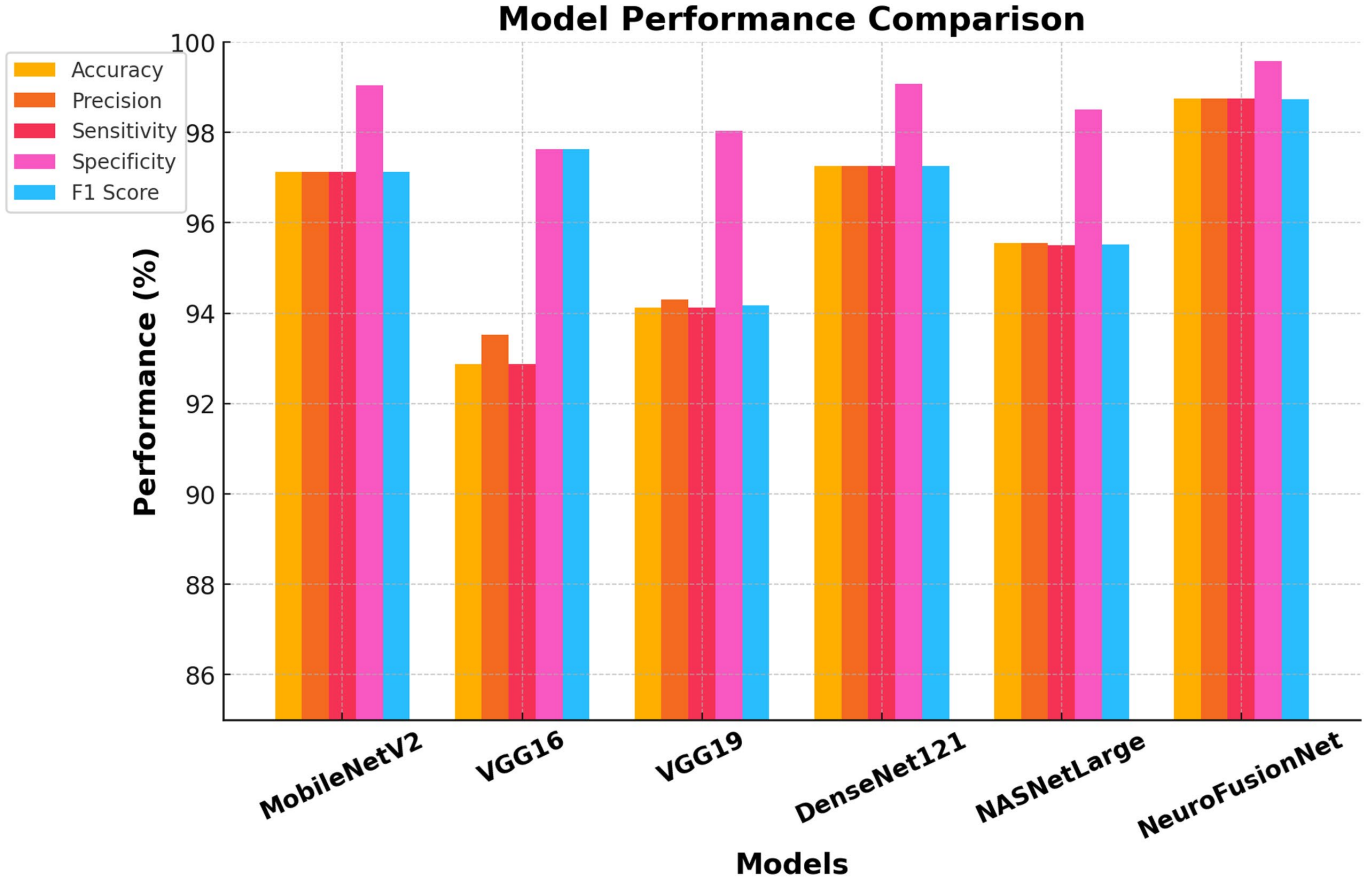
Comparison of performance metrics of different models using the MRI for brain tumor with bounding boxes dataset.

The next step involved using the same pre-trained models on the second dataset, MRI for Brain Tumor with Bounding Boxes, which included three distinct types of tumors and normal tissues. The same hyperparameter settings were used to evaluate each of these models. The proposed model, NeuroFusionNet, was implemented. The model was trained for 50 epochs. Table 4 shows that the accuracy, sensitivity, and specificity achieved values of 98.75, 98.75, and 99.58%, respectively, demonstrating the high ability of NeuroFusionNet to classify brain tumor images. The MobileNetV2 model achieved an accuracy of 97.13%, sensitivity of 97.13%, specificity of 99.04%, and F1 score of 97.12%. The VGG16 model achieves an accuracy of 92.88%. The DenseNet121 and NASNetLarge models showed varying accuracy in classifying brain images, achieving 97.25 and 95.56%, respectively. Figure 13 shows a graphical illustration of the model’s effectiveness.

**Table 4:** Performance evaluation on MRI for brain tumor with bounding boxes dataset.

Model	Accuracy	Precision	Sensitivity	Specificity	F1 score
MobileNetV2	97.13%	97.13%	97.13%	99.04%	97.12%
VGG16	92.88%	93.52%	92.88%	97.63%	93.00%
VGG19	94.13%	94.30%	94.12%	98.04%	94.18%
DenseNet121	97.25%	97.26%	97.25%	99.08%	97.25%
NASNetLarge	95.56%	95.56%	95.50%	98.50%	95.52%
NeuroFusionNet	98.75%	98.75%	98.75%	99.58%	98.74%

**Figure 14:**
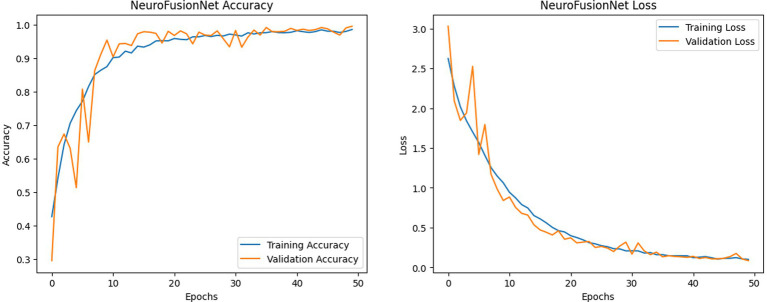
NeuroFusionNet’s training and validation metrics (accuracy and loss) on the MRI for brain tumor with bounding boxes dataset.

**Figure 15:**
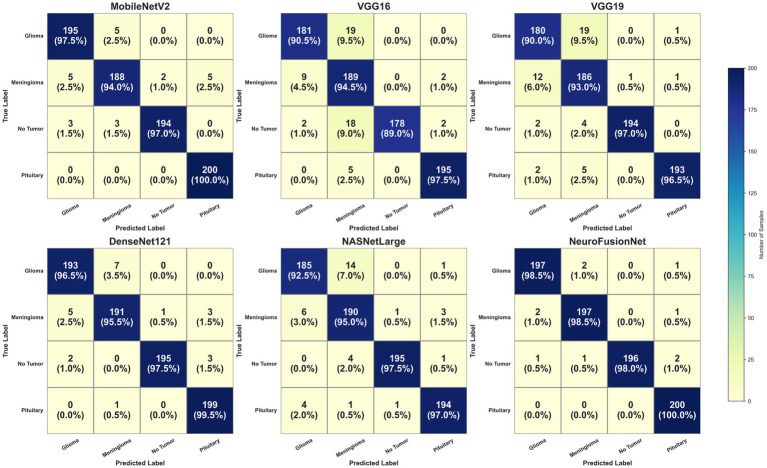
Using the MRI for brain tumor with bounding boxes dataset, the confusion matrices for each model are compared.

From Figure 14, it is noticeable that NeuroFusionNet showed nearly maximally boosted accuracy with a minimal error in training and validation. The model achieved 98.75%. The confusion matrices presented in Figure 15 for five different models, further emphasize the well-documented superiority of NeuroFusionNet due to higher accuracy and fewer incorrect predictions. Confusion matrices summarize the general view of correct and incorrect predictions.

Although we have shown that the proposed NeuroFusionNet is effective on two widely accepted MRI datasets, broader evaluation across clinical sites, modalities, and protocols will be necessary to gather generalizability measures. Future validation efforts of our design will extend to other real datasets such as BraTS, TCGA, and TCIA, where a comparative evaluation will be done to check the overall robustness and actual clinical data adaptability for the model.

To gain further insight into the classification behavior of NeuroFusionNet, we analyzed the confusion matrices (Figures 12, 15) as well as derived class-wise performance metrics. While the overall accuracy almost reaches perfection, examining it closely shows that most wrong predictions are between meningiomas and gliomas. This is because, in some cases, these two types of tumors share certain features of shape and intensity, which makes them indistinguishable on MRI scans. However, as illustrated in the new Supplementary Table S1, the model keeps a very high precision, recall, and F1-score of over 0.97 for all classes, revealing that the model performs well for all classes.

### 3.2 Ethical considerations

Medical diagnosis using artificial intelligence models introduces very serious ethical issues. Of course, first and foremost is patient confidentiality and security of data, especially when handling very sensitive medical information. This study used publicly available datasets. Ethical discussion, therefore, focuses on privacy, bias, transparency, and appropriate clinical use of AI-based decision support. Such measures are necessary to prevent any breach or unauthorized access that may affect the confidentiality of the patients. Furthermore, biases in training data can cascade against AI models, leading to differences in diagnosis accuracy across demographic groups. Continued model validation and testing with multiple populations are needed to alleviate biases and ensure equity in healthcare outcomes.

Artificial intelligence can greatly enhance diagnostic processes; however, it should not replace the judgment of medical professionals. Our approach ensures that AI acts only as a supporting tool and never as a replacement by underlining the need to always retain physician decision-making authority. Emphasizing model interpretability, through which medical practitioners can comprehend and critically question AI recommendations, is of critical importance if trust in AI-assisted diagnosis is to be maintained. Ultimately, this ethical framework is a commitment to the development and implementation of AI technology within health, with the upholding of moral standards.

Nevertheless, present results are very strong, however, future persisting risks of overfitting or unstable training are likely with limited annotated datasets and varied acquisition protocols of MRIs. Current techniques for regularization and validation are effective to an extent, but they could also merit future consideration for dynamic learning rate scheduling. These models in a medical scenario are related to various difficulties regarding integration with hospital systems, real-time inference, and even more transparency in decisions made. One other advanced version of this work in the future would use federated learning and other privacy-preserving techniques, such as differential privacy, that could become highly useful in a very sensitive environment like medical information. NeuroFusionNet achieved high accuracy on the evaluated public datasets; additional external validation and deployment studies are required before clinical use. It can augment radiologists as a second-opinion diagnosis tool, helping them come up with much faster and consistent tumor classification across patients. It’s very small computational appetite allows it to be set up not just on hospital servers, but also on edge devices. By reducing diagnostic time and minimizing human error, the model could improve early detection rates and treatment planning. In moving further into the clinical environment, however, validation on multi-center, real-world datasets and integration with PACS (Picture Archiving and Communication Systems) would be necessary, including all regulatory approvals and development of user-friendly interface.

### 3.3 Limitations of the study

The principal restriction is the necessity for more diverse data to train the model, as inadequate data may compromise the model’s accuracy and effectiveness in addressing atypical events or non-representative datasets. Nevertheless, research data has been well preserved in databases like Kaggle. Nonetheless, there exists a degree of computational complexity. These responsibilities can be mitigated by employing cloud-based software, such as the complimentary edition of Google Colab. Moreover, the concept necessitates considerable computing power, rendering its application difficult in resource-constrained environments. A comprehensive assessment of additional medical imaging modalities, including X-rays and CT scans for diverse conditions, must confirm the model’s efficacy over a broad spectrum of applications. Mitigating these constraints enhances the model’s reliability and comprehensiveness for application in medical practice. Lass imbalance should be assessed directly from class distributions and class-wise performance. Future research will include detailed per-class performance evaluation, including confusion matrices and false positive/negative analysis, to ensure balanced predictive performance across all tumor types, particularly underrepresented ones.

Although the proposed model achieves high accuracy on publicly available datasets, it is important to note that such benchmarks may not fully reflect real-world clinical conditions. Datasets collected from platforms such as Kaggle often exhibit limited variability in imaging protocols, patient demographics, and acquisition settings, which may result in optimistic performance estimates. In contrast, more challenging datasets, such as BraTS, include greater heterogeneity and complexity, providing a more realistic evaluation of model robustness and generalization. Therefore, further validation on diverse and clinically representative datasets is necessary to fully assess the practical applicability of the proposed framework.

## 4 Discussion

This study presents a novel deep learning framework, NeuroFusionNet, for the multiclass classification of brain tumors using MRI images. The model combines a VGG16-based architecture with a custom GAN generator and transfer learning to enhance feature extraction and classification performance. Across two publicly available datasets, the proposed method achieved outstanding classification accuracies of 99.05% and 98.75%, respectively, outperforming several well-established models including VGG16, MobileNetV2, and DenseNet121. These findings demonstrate the model’s potential in improving diagnostic accuracy and efficiency in clinical neuroimaging workflows.

Diagnosis and classification of brain tumors have undergone extensive improvement through the application of deep learning techniques on various medical imaging modalities, such as CT, MRI, and histology. This section presents the most recent progress made from various studies on brain tumors, methods of detection, and multimodal approaches that have reached a high classification rate of benign and malignant brain tumors.

In a study by Mahajan et al. (2025), deep learning was used to scan for brain tumors in MRI images, including both binary and multiclass classification. The study relied on a dataset of 7,753 MRI images, including data harvested from Qhills Technologies Pvt. Ltd. and the Brain Tumor MRI collection. Various models from the deep learning family, such as CNN, AlexNet, and VGG-16, were assessed in terms of performance. VGG-16 gave an accuracy of 96.4%, followed by CNN (73.1%) and AlexNet (76.9%). The methodology included data preprocessing, but there were promising results against many limitations, including the variability in MRI image quality. A study by Lakshmi et al. (2025) suggests another method, Explainable Artificial Intelligence with UNet-based Segmentation and Bayesian Machine Learning for Brain Tumor Classification (XAISS-BMLBT), on MRI-based brain tumor detection. The study could perform the whole analysis concerning four categories using a benchmark MRI dataset of 5,500 samples: glioma, meningioma, pituitary, and no-tumor cases. The methods employed were bilateral filtering, which was performed on the raw images for noise reduction; MEDU-Net + segmentation; feature extraction through ResNet50; and BRANN for classification purposes. Hyperparameter tuning was performed by employing improved radial movement optimization (IRMO) to maximize the performance of the model under study. The record classification accuracy thus achieved was 97.75%, which is superior to that achieved by supervised deep learning models such as ResNet50, Inception-V3, and Fine-tuned VGG19. The study also had limitations and generalization-related concerns regarding real-time applications. Also, the dependence on a single dataset poses additional concerns regarding the capability of the model to perform under different imaging situations and clinical environments.

In study by Pratap Joshi et al. (2025), initiated the Variational Spatial Attention with Graph Convolutional Neural Network (VSA-GCNN) module for brain tumor segmentation and classification using MRI images. The research was mainly focused on the MRI datasets from BraTS 2019, 2020, and 2021. Noise being removed from the images using min-max normalization is an important step before carrying out the segmentation. The proposed methodology integrated tumor segmentation with VSA-GCNN, feature extraction with AlexNet, and classification using Bidirectional Gated Recurrent Unit (Bi-GRU). The results of the experiment indicated that this method had the maximum performance with an accuracy value of 98.2% on the BraTS 2020 dataset. Further, it had relatively high values in sensitivity (99.4%) and specificity (97.6%). Limitations include a well-known dependence on high-quality labeled MRI data and the difficulty in achieving generalizability in the real world; moreover, the variations in MRI acquisition techniques severely affect generalizability. Although it was suggested in the study of Singh et al. (2024), BrainNet, has developed deep learning based on CNN models for the classification of brain tumors using MRI images. The model was developed, trained, and evaluated against well-validated transfer learning architectures such as VGG13, VGG16, VGG19, InceptionResV2, and SqueezeNet. BrainNet had achieved 99.96% percent training accuracy and 97.71% percent test accuracy, higher than those of the comparable models. Limitations were the dependency on large amounts of labeled datasets, computational intensity, and the risk of overfitting with efforts such as dropout and batch normalization, restricting real-world diagnostic uses.

The researcher Alshuhail et al. (2024) presented a deep learning model for brain tumor classification that rendered an accuracy of 98% in four tumor categories. It included image preprocessing steps such as resizing, normalization, and data augmentation. The proposed sequential CNN model involved convolutional, max pooling, dropout, and dense layers along with grad-CAM for interpretability. High accuracy rendered by the model is also dependent on labeled datasets and high computational resources, thus somewhat of a challenge in addition to performance discrepancies caused by differences in demographic data and imaging protocols. A study by Alain Marcel Dikande Simo indicated that an FCNN-based deep learning model performed at a 95% level on multiclass tumor classification (Simo et al., 2024). Using a dataset worth 7,023 images from Kaggle Brain Tumor MRI, this optimized model was trained with Adam and Nesterov momentum optimizers; the latter one proved to be best at balancing precision and recall. The model suffered from limitations like sparsity of the dataset and class imbalance, which limit the generalizability of the model. Further validation and dataset expansion were recommended to make the model more robust.

In their study, Muhammad Sami Ullah proposed BrainNet - a deep learning framework with residual blocks and stacked autoencoders for multimodal classification of a brain tumor (Ullah et al., 2024). This framework, which was trained using the BraTS2020 and BraTS2021 datasets, consists of a stacked encoder-decoder network of 10 convolutional layers along with a modified Grey Wolf Optimization technique to achieve 98% accuracy. However, it also involved very high computational costs, hyperparameter searches across large landscapes, and the complexities of carrying over such models for a very diverse set of real-world datasets. In their recent research (Reyes and Sánchez, 2024), Daniel Reyes and Javier Sánchez conducted a thorough comparative study of several normalized CNNs for brain tumor classification. VGG, ResNet, EfficientNet, MobileNet, and ConvNeXt were a few of the architectures evaluated against two MRI datasets. The best performance of these models attained a stunning accuracy of 98.7%, illustrating rather sane results. There were also mentions of drawbacks such as heft computational costs, long training times, and limited data augmentation advantages. Krishnan also devised a Rotation Invariant Vision Transformer (RViT) model (Krishnan et al., 2024) in his research, which urges improving classification accuracy using fused rotated patch embeddings to address tumor orientation issues in MRIs. With 98.6% accuracy and high sensitivity and specificity, the model was evaluated on the Kaggle Brain Tumor MRI Dataset. Its only limitation in fall consumption is that it can only work on binary classification, which is a limiting factor for many in multi-class tumor detection; on top of that, additional computational complexity poses a challenge to its clinical deployment.

Unlike previous studies that primarily rely on standalone CNN architectures or use GANs solely for data augmentation, the proposed NeuroFusionNet introduces a hybrid learning paradigm where the GAN component actively contributes to feature representation learning. While transformer-based models have shown promising results, they often require large-scale datasets and high computational resources, which limits their applicability in medical imaging. In contrast, NeuroFusionNet achieves superior performance by leveraging a lightweight yet effective architecture combined with adversarial feature enhancement. Furthermore, the proposed model demonstrates consistent performance across multiple datasets, addressing the generalization limitations observed in prior works. As shown in Table 5.

**Table 5:** Systematic comparison of existing deep learning models for brain tumor classification.

Study	Architecture	Dataset	Accuracy	Limitation	Difference from NeuroFusionNet
Mahajan et al. (2025)	VGG16, CNN	7,753 MRI	96.4%	Limited robustness	No GAN integration
Lakshmi et al. (2025)	UNet + ResNet50	5,500 MRI	97.75%	Single dataset	No feature-level GAN learning
Pratap Joshi et al. (2025)	VSA-GCNN + BiGRU	BraTS	98.2%	High data dependency	Complex pipeline
Singh et al. (2024)	CNN variants	MRI dataset	97.71%	Overfitting risk	No adversarial learning
Alshuhail et al. (2024)	CNN	MRI dataset	98%	Data dependency	No GAN/limited generalization
Simo et al. (2024)	FCNN-based deep learning	Kaggle Brain Tumor MRI	95%	The sparsity of the dataset	No GAN/limited generalization
Ullah et al. (2024)	Autoencoder + Residual	BraTS	98%	High computational cost	No feature-level fusion
Reyes and Sánchez (2024)	VGG, ResNet, EfficientNet, MobileNet, and ConvNeXt	MRI dataset	The best performance of these models attained accuracy of 98.7%	heft computational costs	No GAN/limited generalization
Krishnan et al. (2024)	Vision transformer	MRI	98.6%	Binary only	No multiclass generalization
Proposed model	VGG16 + GAN	Multiple datasets	99.05%	—	Feature-level GAN integration

## 5 Conclusion and further work

In conclusion, NeuroFusionNet performed strongly for four-class brain MRI classification on the evaluated public datasets. We can efficiently employ synthetic feature augmentation and high-level representations from medical imaging data by combining a pre-trained VGG16 model with generative adversarial networks (GANs) for feature extraction and transfer learning. The model employs data augmentation methods, fine-tuning approaches, and an optimal learning rate schedule to enhance generalization and robustness. In the Brain Tumor MRI dataset, NeuroFusionNet achieves the best accuracy of 99.05% in MRI scan analysis, whereas it attains 98.75% accuracy utilizing the Bounding Boxes Dataset for brain tumors.

Future work aims to improve the model’s reliability and generalizability by applying k-fold cross-validation and testing it on diverse, external datasets from independent institutions. It will also involve a deeper analysis of brain cancer subtypes using larger datasets that consider age and gender differences. Advanced techniques such as digital twins and federated learning will be integrated to enhance real-world applicability. Additionally, performance will be improved through modern architectures like Transformers, Vision Transformers (ViTs), spatial attention mechanisms, and hybrid CNN–RNN models for better feature extraction from MRI scans. Overall, these enhancements are expected to increase accuracy, interpretability, and robustness beyond the already strong current results.

## Data Availability

The original contributions presented in the study are included in the article/supplementary material, further inquiries can be directed to the corresponding author.
